# ACCELERATED AGING AMONG ADULTS LIVING WITH CEREBRAL PALSY

**DOI:** 10.1093/geroni/igac059.515

**Published:** 2022-12-20

**Authors:** Mark Peterson, Sudarshan Dayanidhi, Patrick McPhee, Heidi Haapala

**Affiliations:** University of Michigan, Ann Arbor, Michigan, United States; Shriley Ryan AbilityLab/Northwestern University, Chicago, Illinois, United States; McMaster University, Hamilton, Ontario, Canada; University of Michigan, Ann Arbor, Michigan, United States

## Abstract

Cerebral Palsy (CP) is the most common pediatric-onset physical disability, with an estimated prevalence ranging from 2.6-3.1 cases per 1,000 live births in the United States. There is a lack of clinical follow-up for individuals with CP after they transition from pediatric to adult primary care, and insufficient surveillance to track patients with CP longitudinally. Despite the shortage of research to examine the natural history of CP and chronic disease trajectories in this population, a range of secondary conditions arise at an accelerated rate as compared to the adult population without CP, prompting the widespread notion and clinical hypothesis that patients with CP are prone to accelerated aging. These factors further worsen functional status and quality of life, as well as lead to decreased independence. Despite the well-established interrelationships between physical and mental health disorders in the non-CP older adult population, the extent to which, mechanisms underlying, and time course associated with the development of these chronic conditions among adults living with CP has received little empirical attention. The proposed course will build upon our ongoing work by highlighting new findings from three centers doing research pertaining to aging with CP, and will cover novel mechanisms of musculoskeletal pathophysiology in CP, risk factors of and unique CVD profiles among adults with CP, and new findings related to health trajectory differences of adults with CP from clinical and population-representative cohorts. We will also provide insights into the pathophysiologic mechanisms linking early frailty and long-term health outcomes among persons with CP.

